# An Improved Method of RNA Isolation from Loblolly Pine (*P. taeda* L.) and Other Conifer Species

**DOI:** 10.3791/1751

**Published:** 2010-02-22

**Authors:** W. Walter Lorenz, Yuan-Sheng Yu, Jeffrey F. D. Dean

**Affiliations:** Warnell School of Forestry and Natural Resources, University of Georgia (UGA)

## Abstract

Tissues isolated from conifer species, particularly those belonging to the Pinaceae family, such as loblolly pine (*Pinus taeda* L.), contain high concentrations of phenolic compounds and polysaccharides that interfere with RNA purification.  Isolation of high-quality RNA from these species requires rigorous tissue collection procedures in the field and the employment of an RNA isolation protocol comprised of multiple organic extraction steps in order to isolate RNA of sufficient quality for microarray and other genomic analyses.  The isolation of high-quality RNA from field-collected loblolly pine samples can be challenging, but several modifications to standard tissue and RNA isolation procedures greatly improve results.  The extent of general RNA degradation increases if samples are not properly collected and transported from the field, especially during large-scale harvests.  Total RNA yields can be increased significantly by pulverizing samples in a liquid nitrogen freezer mill prior to RNA isolation, especially when samples come from woody tissues.  This is primarily due to the presence of oxidizing agents, such as phenolic compounds, and polysaccharides that are both present at high levels in extracts from the woody tissues of most conifer species.  If not removed, these contaminants can carry over leading to problems, such as RNA degradation, that result in low yields and a poor quality RNA sample.  Carryover of phenolic compounds, as well as polysaccharides, can also reduce or even completely eliminate the activity of reverse transcriptase or other polymerases commonly used for cDNA synthesis.  In particular, RNA destined to be used as template for double-stranded cDNA synthesis in the generation of cDNA libraries, single-stranded cDNA synthesis for PCR or qPCR's, or for the synthesis of microarray target materials must be of the highest quality if researchers expect to obtain optimal results.  RNA isolation techniques commonly employed for many other plant species are often insufficient in their ability to remove these contaminants from conifer samples and thus do not yield total RNA samples suitable for downstream manipulations.  In this video we demonstrate methods for field collection of conifer tissues, beginning with the felling of a forty year-old tree, to the harvesting of phloem, secondary xylem, and reaction wood xylem.  We also demonstrate an RNA isolation protocol that has consistently yielded high-quality RNA for subsequent enzymatic manipulations.

**Figure:**
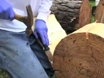


## Protocol

### Part 1: Tree Harvest

*After the tree is selected and felled, it is important to work as carefully and as quickly as possible.  As each different tissue type is harvested, they should be placed immediately into their own liquid nitrogen vessels to avoid any cross contamination of sample material.  This RNA isolation protocol is scalable.*

1. Cut bolts into lengths of approximately 0.5-0.75 m and with a chainsaw score the bark in two or three places following the longitudinal axis of the bolt.
2. Using a wood chisel, work the edge of the scored region until the bark begins to separate from the wood and continue working the chisel while pulling back on the section of bark until it separates completely from the stem.
3. Secondary xylem is harvested with a straight edge, single sided razor by scraping along the longitudinal axis of the bolt that has been stripped of bark. Wear latex gloves to minimize contamination of the tissue.
4. Phloem, which is located on the inner side of the bark sections, is readily harvested by manual peeling.
5. Reaction wood is collected from the underside of limbs after marking them with spray paint to indicate their original orientation with respect the ground.
6. Limbs are cut into bolts of 1-2 m and scored on opposite sides along the longitudinal axis according to the approximate locations of the two types of reaction wood: opposite wood (on the top side of the limb) and compression wood (on the bottom side of the limb).  The bark is separated from the limb as described above, except that only the bark covering each type of reaction wood is removed at one time.  Secondary xylem or phloem is then collected as described above and placed in liquid nitrogen.
7. Other samples, such as needles, shoot tips and young cones, can be harvested by hand or using pruning shears as required.
8. Collected samples frozen in liquid nitrogen should be placed in marked bags or suitable containers and packed in dry ice for shipment back to the laboratory.

### Part 2: Freezer Mill Processing of Samples

1. Fill the Spex 6850 freezer mill reservoir with liquid nitrogen and place the impact bar into one of the grinding vials and fill with liquid nitrogen to pre-chill.  Pour off liquid nitrogen and add sample to vial.
2. Make sure that no excess liquid nitrogen remains in the milling chamber and that excess nitrogen gas is completely vented before the end cap is seated to seal the chamber.
3. Insert the grinding vial into the carrier and close.
4. Mill woody samples for two cycles of 1 minute/cycle at a rate setting of 14.
5. Use a liquid nitrogen chilled spatula to remove the milled sample (wood flour) into a beaker that has been pre-chilled and contains a small amount of liquid nitrogen.
6. Pour the liquid nitrogen/sample slurry into a storage container and place at -80° C until needed.

### Part 3: RNA Isolation, Day 1

1. Place 20mL of RNA Isolation Buffer into a 50mL capped Falcon tube and add 400 μL β-mercaptoethanol, vortex briefly, then heat in a 60° C water bath.
2. Add 4 g of frozen tissue to each tube. Cap and shake vigorously by hand followed by vortexing for 10 seconds.
3. Add an equal volume of chloroform to the tube and invert gently to mix. Place the tube on a rotating wheel and allow end-over-end mixing for 5 minutes.
4. Decant the mixture into a 50 mL Oak Ridge tube and centrifuge at 12,000 (17,200 xg) rpm in a SS34 fixed-angle rotor for 5 minutes.
5. Transfer the aqueous phase to a fresh Oak Ridge tube and add a half-volume of chloroform.
6. Vortex for 1 minute and continue mixing on a rotating wheel for 5 minutes. Centrifuge at 12,000rpm (17,200 xg) in a SS34 fixed angle rotor for 5 minutes.
7. Transfer the aqueous phase to fresh Oak Ridge tube and add a half-volume of chloroform. Vortex for 1 minute. Centrifuge at 12,000 rpm (17,200 xg) in a SS34 fixed angle rotor for 10 minutes.
8. Transfer the aqueous phase to a 50 mL high speed polypropylene tube. Centrifuge at 10,000 rpm (11,900 xg) in a SS34 fixed angle rotor for 20 minutes.
9. Decant supernatant into 50 mL Falcon tube and add a quarter-volume 10 M LiCl solution to supernatant. Briefly vortex and place at 4° C for overnight precipitation.

### Part 4: RNA Isolation, Day 2

1. Heat SSTE buffer in a 65° C water bath prior to starting.
2. Pour the LiCl-precipitated sample into a polypropylene tube and pellet the RNA by centrifugation at 11,000rpm in a SS34 fixed angle rotor (14,450 xg) for 30 minutes at 4° C.
3. After centrifugation, pour off and discard the liquid, and invert the tubes on Kimwipes for approximately 1 minute.  Pipette off any remaining supernatant.
4. Resuspend each pellet in 800 μL of heated SSTE using a pipette to thoroughly mix the samples. Transfer the resuspended sample to 2 mL Ambion RNAse-free microfuge tubes.
5. Heat samples at 65° C for 2 minutes followed by vigorous vortexing to ensure complete resuspension.
6. Add an equal volume of phenol-chloroform, pH8, to each sample tube. Vortex each sample for 30 seconds and then spin in a microfuge at 14,000 rpm for 3 minutes
7. Transfer the aqueous phase to a new 2mL Ambion RNAse-free microfuge tubes, and add an equal volume of chloroform.  Vortex each sample for 30 seconds and spin in the microfuge at 14,000rpm for 3 minutes.
8. Transfer aqueous phase to Phase-Lock Gel tubes that have been previously prepared by microfuging at 11,000 rpm for 2 minutes. Add a half-volume of chloroform and gently pipette to mix.
9. Centrifuge at 11,000 rpm for 5 minutes and transfer the aqueous phase to fresh 2mL Ambion RNAse-free microfuge tubes.

### Part 5: RNA Precipitation and Ethanol Wash

1. Add 50 μL 3.0 M sodium acetate, pH 4.8, and 1 mL 100% ethanol to the aqueous phase. Vortex and precipitate in -80° C for 30 minutes or overnight at -20° C.
2. Microfuge samples at 14,000 rpm for 30 minutes at 4° C, and carefully aspirate off the supernatant. Do not disturb the pellet.
3. Wash the RNA pellets with 1 mL -20° C 70% (v/v) ethanol.  Microfuge at 14,000 rpm for 1 minute and aspirate off the ethanol.
4. Repeat step 5.3.
5. Uncap the tubes and air-dry pellets on the bench for 3-5 minutes.

### Part 6: Final Resuspension and Quantification

1. Resuspend each pellet in 100 μL DEPC-treated water. Mix by vortexing and incubate the tubes at 65° C for 2 minutes before placing on ice.  Repeat if necessary to ensure sample resuspension.
2. Pool like samples if desired and make 50 μL of a 1:10 dilution in 10mM Tris, pH 7.5, for spectrophotometric quantitation.  Absorbance ratios for 260/280 and 260/230 are typically greater than 2.1 and 2.2, respectively.  RNA isolation yields range from 60-120μg/g frozen tissue.
3. Analyze samples by running 1.5-2.5 μg RNA on a 1% agarose gel in TAE buffer.  For more critical samples, analyze the RNA using an Agilent 2100 Bioanalyzer as per the manufacturer's instructions.

### Representative Results:

 RNA isolation yields from different conifer tissues range from 60-120 μg/g frozen tissue with woody samples typically yielding around 70-80g/g frozen tissue.  Diagnostic ratios of 260/280 and 260/230 are both typically greater than 2.1, and are good indicators that the RNA sample is free of any significant phenolic or carbohydrate contamination, respectively.  Samples analyzed by agarose gel electrophoresis followed by EtBr staining should show three distinct bands for 25S, 18S, and 5S RNA (Figure 1).  Determining the stoichiometry of the 28S to 18S on agarose gels is somewhat subjective, and factors such as running conditions for the gel, staining, and sample load can all have an effect.  In general, the 28S:18S ratio will appear to be > 1.5.  However, some conifer samples have lower ratios.  For example, following Agilent 2100 analysis we have seen several instances of conifer samples from different tissue types where the stoichiometry is closer to 1.2:1.  Thus, an apparent low 28S:18S stoichiometry by agarose gel electrophoresis does not necessarily indicate a poor-quality sample. Leaf, shoot, and cone samples will often have additional distinct bands present due to chloroplastic ribosomal RNAs.  EtBr-stained materials that do not migrate from the sample well or high molecular mass bands (>8-10 kb) usually indicate genomic DNA contamination (Figure 2).  Low molecular mass smears in the vicinity of or below the 5S band and reduced ribosomal band staining or an apparent 18S > 28S stoichiometry is a good indicator that RNA degradation has occurred (Figure 3).  In the Agilent 2100 electropherogram analysis, the base line should be low and relatively smooth with major peaks corresponding to 18S and 28S ribosomal RNAs having 28S:18S ratios ranging anywhere from 1.2 to 2.0.  The Agilent RNA Integrity Number (RIN) value may be a better predictor of RNA quality and should be at least 7 or greater for RNA that is to be used for the preparation of microarray targets.  Figure 4 shows a typical Agilent 2100 electropherogram for xylem RNA with a 28S:18S ratio equal to 1.4 and a RIN value of 8.6 while Figure 5 shows a poor xylem RNA prep having a very high baseline and noticeable degradation as revealed by the 28S:18S ratio equal to 0.65 and a RIN value of 3.4.


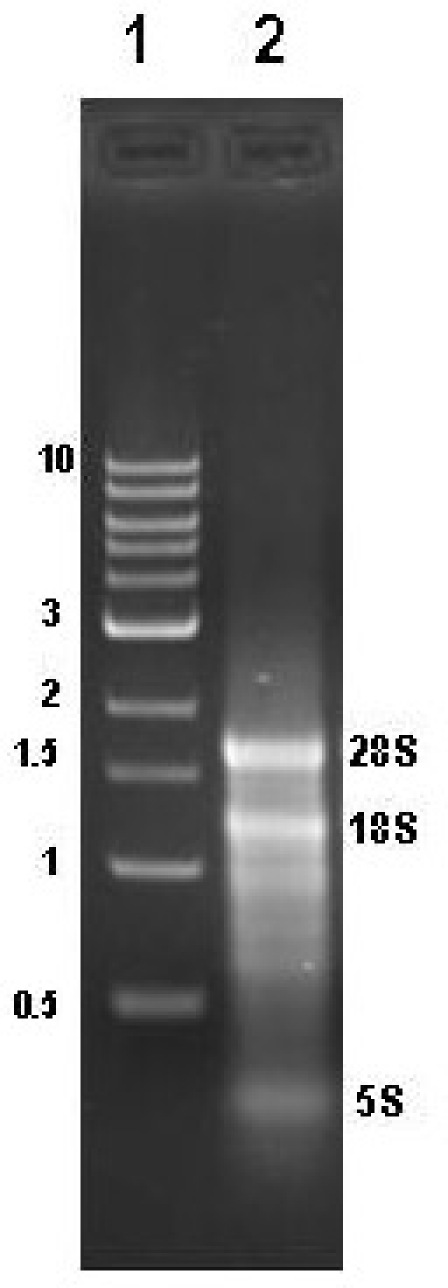
 **Figure 1.**  Agarose gel analysis of high-quality pine RNA. Lane 1, NEB 1 kb standard, Lane 2 shows a typical total RNA isolation from loblolly pine secondary xylem with characteristic ribosomal 28S, 18S, and 5S bands and apparent 28S:18S ratio >1.


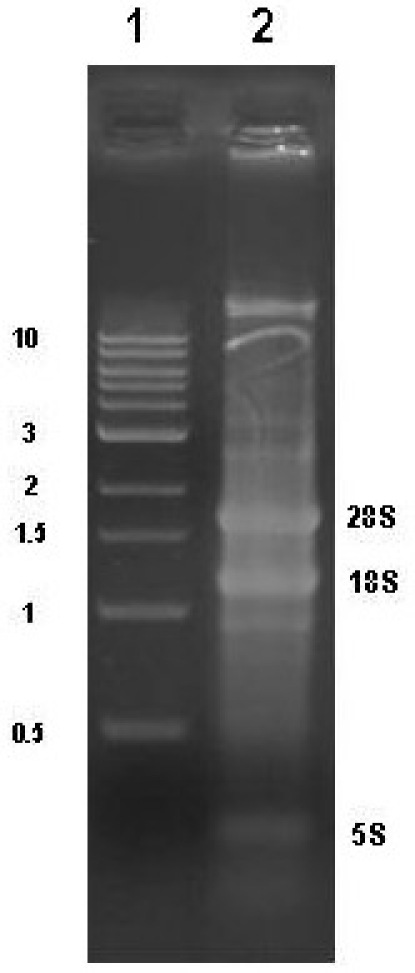
 **Figure 2.**Agarose gel analysis of pine RNA sample contaminated with genomic DNA. Lane 1, NEB 1 kb standard, Lane 2, xylem RNA sample showing genomic DNA contamination.


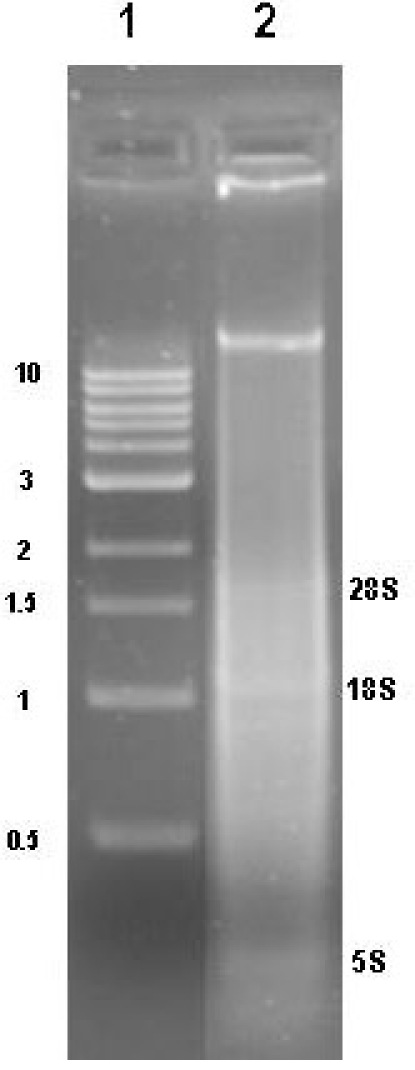
 **Figure 3.**Agarose gel analysis of pine sample showing RNA degradation and contamination with genomic DNA. Lane 1, NEB 1 kb standard, Lane2, xylem RNA sample showing genomic DNA contamination and severe RNA degradation.


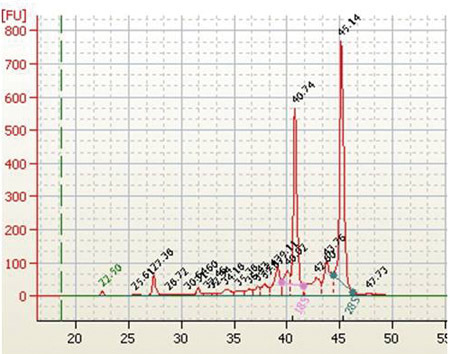
 **Figure 4.**Agilent 2100 electropherogram of xylem total RNA isolated using the described method on loblolly pine xylem.  28S:18S ratio equals 1.4, RIN value equals 8.6 


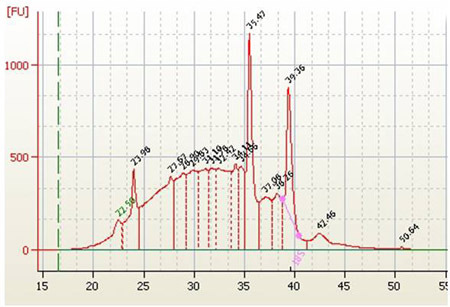
 **Figure 5.**Agilent 2100 electropherogram of apical tip total RNA showing severe degradation and genomic contamination.   28S:18S ratio equals 0.65, RIN value equals 3.4 

## Discussion

Obtaining high-quality RNA from conifer species can be a difficult task given the high levels of phenolic and polysaccharide compounds found in woody tissues.  Starting with the protocol developed by Chang et al. (1), we have found that more rigorous extraction and clean up steps lead to the consistent isolation of very high-quality total RNA from various woody and non-woody tissues sampled from a wide variety of conifer species.  This video has demonstrated not only our modified RNA isolation protocol, but also the steps taken in field collection and processing techniques used for loblolly pine prior to RNA isolation.  These harvesting and preparation steps are equally important to minimize RNA degradation in field-collected samples, as well as to increase both RNA quality and total yield.  Most significantly, we have found that RNA prepared using this method has led to increased cDNA synthesis yields compared to other methods used to prepare microarray targets for hybridization against the PtGen2 loblolly pine microarray (2).
